# Using technology to deliver cancer follow-up: a systematic review

**DOI:** 10.1186/1471-2407-14-311

**Published:** 2014-05-03

**Authors:** Rebekah Dickinson, Susan Hall, Jenny E Sinclair, Christine Bond, Peter Murchie

**Affiliations:** 1 Wednesbury Malling Health Practice, High Bullen, Wednesbury, West Midlands WS10 7HP, UK; 2 Division of Applied Health Science, Centre of Academic Primary Care, University of Aberdeen, Polwarth Building, Foresterhill, Aberdeen AB25 2ZD, Scotland, UK

## Abstract

**Background:**

People with cancer receive regular structured follow up after initial treatment, usually by a specialist in a cancer centre. Increasing numbers of cancer survivors prompts interest in alternative structured follow-up models. There is worldwide evidence of increasing interest in delivering cancer follow-up using technology. This review sough evidence supporting the use of technology in cancer follow-up from good quality randomised controlled trials.

**Method:**

A search strategy was developed to identify randomised controlled trials and reviews of randomised trials of interventions delivering some aspect of structured cancer follow-up using new technologies. Databases searched were: All EBM Reviews; Embase; Medline (No Revisions); Medline (Non-Indexed Citations), and CAB Abstracts. Included articles were published in English between 2000 and 2014. Key words were generated by the research question. Papers were read independently and appraised using a standardised checklist by two researchers, with differences being resolved by consensus [*J Epidemiol Community Health*, **52**:377–384, 1998]. Information was collected on the purpose, process, results and limitations of each study. All outcomes were considered, but particular attention paid to areas under consideration in the review question.

**Results:**

The search strategy generated 22879 titles. Following removal of duplicates and abstract review 17 full papers pertaining to 13 randomised controlled studies were reviewed. Studies varied in technologies used and the elements of follow-up delivered, length of follow-up, tumour type and numbers participating. Most studies employed only standard telephone follow-up. Most studies involved women with breast cancer and included telephone follow-up. Together the results suggest that interventions comprising technology had not compromised patient satisfaction or safety, as measured by symptoms, health related quality of life or psychological distress. There was insufficient evidence to comment on the cost effectiveness of technological cancer follow-up interventions.

**Conclusions:**

Modern technology could deliver cancer follow-up that is acceptable and safe. More research is required to develop cancer follow-up systems which exploit modern technology, which should be assessed using randomised trials, with consistent outcomes, so that evidence on the acceptability, safety, cost effectiveness and impact in quality of life of technological follow-up can accumulate and be made available to patients, professionals and policy makers.

## Background

Following the completion of their primary treatment for cancer most patients enter a programme of structured follow-up [1,2]. This is usually based in secondary care and involves regular face to face consultations with specialist cancer doctors; the precise frequency and content of follow-up visits varies according to cancer site and local and national guidelines [3]. Follow-up care is generally focused on detecting recurrent disease, monitoring the effects of treatment and providing ongoing support to patients and their families and there is good evidence that such care is valued by patients [4].

Current models of cancer follow-up are likely to be unsustainable due to two important factors. Firstly, as the population ages and treatment improves cancer prevalence increases year on year [5]. This means that secondary care services are tasked with the delivery of follow-up to an increasing number of patients, and generally without corresponding increase in resources [6]. Secondly, accessing aftercare can be problematic for certain patient groups, especially those who live in remote and rural areas distant from cancer centres [3]. Access difficulties to cancer follow-up care could be one reason for the observation in some areas of poorer outcomes in rural, compared to urban, cancer patients [7]. In contrast modern technology develops apace and offers increasing capability and functionality to patients, professionals and health systems for care delivery. Furthermore, the current population of cancer patients are increasingly familiar with technology and consuming healthcare information and services on digital platforms.

These issues together are the drivers to develop modern and alternative models of cancer follow-up. To date these have included varying the person delivering care (e.g. a specialist nurse rather than a doctor) and varying the location of cancer follow-up delivery (e.g. primary rather than secondary care) [4]. Models of care that have been subjected to randomised trials have included shared care, nurse-led follow-up and GP-led follow-up, as well as shifting the locus of care from hospital to the community [4]. A further alternative is to exploit the current innovative technological environment and seek to understand how digital means may be employed to deliver some or all aspects of cancer follow-up care [8].

“Telemedicine” is defined as using technology to share information over a distance between healthcare providers (e.g. between GPs and hospital specialists), whereas “telehealthcare” is defined as using technology to provide personalised healthcare to patients at a distance [9,10]. To date some investigators have incorporated the use of land-line and mobile telephones into the delivery of cancer follow-up [11-13].

As technology improves and cancer prevalence increases interest in developing models of follow-up care that employ novel technologies is certain to increase, for example monitoring chemotherapy effects using smartphones [12]. This systematic review was conducted to evaluate existing evidence on the clinical safety, patient acceptability, cost effectiveness and impact on quality of life from telemedicine and telehealthcare where it has been applied to cancer follow-up.

## Methods

### Search and identification of studies

The population of interest was adults with cancer. The intervention was cancer follow up using technology and the control usual care. Inclusion criteria were randomised controlled studies published in English between 2000 and 2014, whose intervention included a telemedicine or telehealthcare element in the intervention. Studies not meeting these criteria were excluded. The key outcomes of interest were patient acceptability (satisfaction), clinical safety and cost effectiveness.

A search strategy based on key words to reflect the review aim was designed in conjunction with a medical librarian and is included as Additional file 1.

The searches were run in February 2014 on the following databases: All EBM Reviews; Embase; Medline (No Revisions); Medline (Non-Indexed Citations); CAB Abstracts. Retrieved citations were exported to Refworks (http://www.refworks.com). All identified titles were read and those not meeting the inclusion criteria were excluded, as were duplicates. Abstracts of the remaining studies were screened against the inclusion criteria and full articles were then retrieved.

### Data collection

Critical appraisal of selected studies was undertaken using a standardized checklist [14]. This was done by two researchers (RD and SH). There was subsequent discussion of assigned scores, and discussion and resolution of any differences by consensus. Papers were analyzed thematically considering particularly outcomes that related to themes highlighted in the review question, namely, clinical safety, patient acceptability, cost effectiveness and impact on quality of life. Clinical safety was defined as any outcome related to recurrent cancer or mortality. Patient acceptability was defined as outcomes reflecting how easily patients had found engaging with an intervention, the extent to which it met their healthcare needs, or how it impacted upon their satisfaction with services. Cost effectiveness related to the reporting of appropriate economic data. Quality of life related to any outcomes reflecting symptoms or accepted or validated measures of health related quality of life. Information was collected on the purpose, process, results and limitations of each study using a standardised data collection template. All outcomes were considered with particular attention paid to issues of patient acceptability and satisfaction, clinical safety, cost and impact on quality of life. Where quantitative data was presented it was tabulated as p values, confidence intervals and effect sizes. A narrative analysis of all papers was also conducted to identify the emergent common and contrasting themes from the reported studies.

## Results

### Study selection

**Figure 1:**
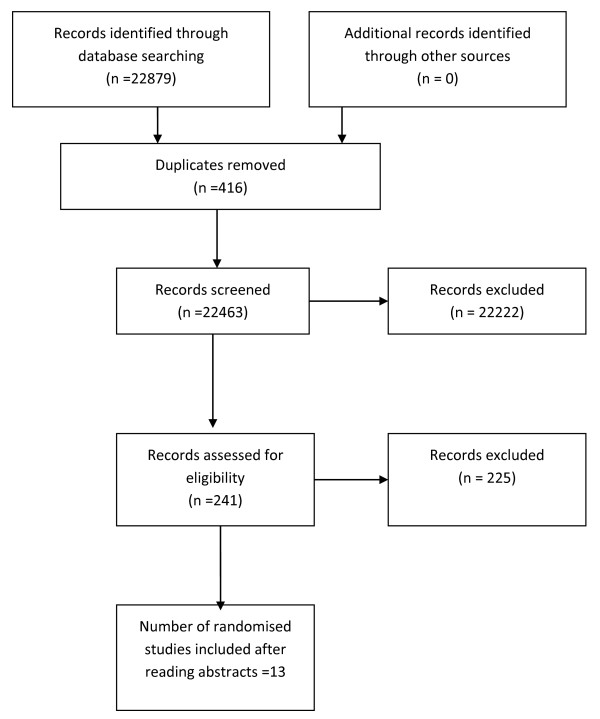
PRISMA 2009 flow diagram.

Figure 1, a PRISMA diagram, displays the data for the number of titles initially identified, then excluded along with duplicates and the final number of randomised studies identified and included.

### Characteristics of included studies

Seventeen papers pertaining to 13 randomised studies were included in the review. Tables 1 and 2 considers and displays each of these studies in detail including terms of patient population, intervention, outcome measures, key results, study quality score, measures used and detailed results including effect sizes.

**Table 1:** Details of included studies including critical appraisal scores

**Study and location**	**Patient population**	**Intervention**	**Control**	**Length of follow up**	**Study outcomes**	**Results**	**Critical appraisal score**
Beaver et al., 2009 Manchester, UK [11]	374 breast cancer patients	Telephone follow up by specialist nurses	Usual hospital care	24 months (mean)	Psychological morbidity	Equivalence trial - : no difference between the two groups	Study Quality – 8/10
					Participant’s needs for information		External validity – 2/3
					Participant’s satisfaction		Internal Validity (bias) – 6/7
					Clinical Investigations ordered		Internal Validity (selection bias) – 6/6
					Time to detection of recurrent disease		Power – 1/1 (Total – 23/27)
Beaver et al., 2009 (Economic evaluation) Manchester, UK [25]	374 breast cancer patients	Cost minimization analysis of RCT above	-	24 months (mean)	Primary: NHS resource use	Telephone follow-up more costly (mean difference £55 but telephone patients had lower personal costs (mean difference £47)	No score as cost analysis
					Secondary: patient, carer and productivity courses		
Davison and Degner, 2002 Vancouver, Canada [15]	749 breast cancer patients	Computer programme providing information and assisting decision making	Standard care only- asked about decision making before clinic appointment	One clinic visit	Involvement in decision making	Women in the intervention group reporting playing a more passive role.	Study Quality – 6/10
					Patient satisfaction	Patient satisfaction was high in both groups	External validity – 2/3
							Internal Validity (bias) – 5/7
							Internal Validity (selection bias) – 4/6
							Power – 0/1 (Total – 17/27)
Harrison et al., 2011 Sydney, Australia [21]	75 patients with colorectal cancer	5 telephone calls from a specialist colorectal nurse in 6 months after discharge	Standard care	6 months	Unmet supportive care needs	No difference between the groups for unmet needs and health service utilization	Study Quality – 8/10
					Health service utilization	Quality of life scores higher in the intervention group at 6 months	External validity – 2/3
					Quality of life		Internal Validity (bias) – 5/7
							Internal Validity (selection bias) – 6/6
							Power – 0/1 (Total – 21/27)
Hegel et al., 2010 New Hampshire, USA [16]	31 Breast cancer patients	6 weekly session of telephone delivered problem solving occupational therapy	Usual care	12 weeks	Primary outcome: feasibility of conducting the trial	Overall positive outcomes	Study Quality – 8/10
					Secondary outcomes: functional, quality of life and emotional status		External validity – 3/3
							Internal Validity (bias) – 5/7
							Internal Validity (selection bias) – 6/6
							Power – 0/1 (Total – 20/20)
Kearney et al., 2008 Stirling, Scotland [12]	112 cancer patients	Mobile phone-based remote monitoring during chemotherapy	Standard care	16 weeks	Chemotherapy related morbidity – 6 common symptoms, nausea, vomiting, fatigue, mucositis, hand-foot syndrome and diarrhoea	Higher reports of fatigue in the control group and lower reports of hand-foot syndrome in the control group	Study Quality – 8/10
							External validity – 1/3
							Internal Validity (bias) – 5/7
							Internal Validity (selection bias) – 6/6
							Power – 0/1 (Total – 20/27)
Kimman et al., 2011 Maastricht, Netherlands [17]	299 women with breast cancer	Nurse led telephone follow up or	Hospital follow up or hospital follow up plus EGP	18 months	Health related quality of life (HRQoL)	No difference between the two groups	Study Quality – 8/10
		Nurse led telephone follow up plus educational group programme (EGP)			Secondary measures included role and emotional functioning and feelings of control and anxiety		External validity – 2/3
							Internal Validity (bias) – 5/7
							Internal Validity (selection bias) – 6/6
							Power – 1/1 (Total – 22/27)
Kimman et al., 2011 Maastricht, Netherlands [27]	299 women with breast cancer	Nurse led telephone follow up or Nurse led telephone follow up plus educational group programme (EGP)	Hospital follow up or hospital follow up plus EGP	18 months	Quality adjusted life gain (QALYs)	Hospital follow-up plus EGP resulted in the highest QALYs but has the highest costs. Next best in terms of costs and QALYs was nurse led telephone follow up plus EGP	No score as cost analysis
					Incremental cost-effectiveness ratios (ICERs)		
Kimman et al., 2010 Maastricht, Netherlands [13]	299 women with breast cancer	Nurse led telephone follow up or	Hospital follow up or hospital follow up plus EGP	12 months	Patient satisfaction	Increased patient satisfaction with access to care in telephone follow-up group. No significant influence on general patient satisfaction, technical competence or inter-personal aspects	Study Quality – 9/10
		Nurse led telephone follow up plus educational group programme (EGP)					External validity – 2/3
							Internal Validity (bias) – 5/7
							Internal Validity (selection bias) – 5/6
							Power – 1/1 (Total – 22/27)
Kroenke et al., 2010 Indiana, USA [26]	405 cancer patients	Centralized telecare management by a nurse-physican specialist team coupled with home-based symptom monitoring by interactive voice recording or internet	Usual care	12 months	Depression Pain	Improvements in pain and depression for the intervention group	Study Quality – 8/10
							External validity – 2/3
							Internal Validity (bias) 6/7-
							Internal Validity (selection bias) – 6/6
							Power – 1/1 (Total – 23/27)
Marcus et al., 2009 Colorado, USA [18]	304 breast cancer patients	16 session telephone counselling post treatment	Resource directory for breast cancer was given to each patient	18 months	Distress	No difference for distress and depression	Study Quality – 8/10
					Depression	Need for clinical referral – depression and distress reduced by 50% in the intervention group for dichotomized end points	External validity – 2/3
					Sexual dysfunction	Effects found for personal growth and sexual dysfunction in the intervention group	Internal Validity (bias) – 5/7
					Personal growth		Internal Validity – 5/6 (selection bias)
							Power – 0/1 (Total – 20/27)
Matthew et al., 2007 Toronto, Canada [20]	152 prostate cancer patients	PDA survey followed by paper	Paper followed by PDA survey	30 mins	Survey was monitoring health-related quality of life but outcomes looked at assessment of data quality and feasibility	Internal consistency similar	Study Quality – 8/10
		PDA followed by PDA survey. (3 groups)				Test re-test reliability confirmed	External validity – 3/3
						Data from two modalities strongly correlated.	Internal Validity (bias) – 5/7
						Fewer missed items for the PDA	Internal Validity (selection bias) – 5/6
						More preferred using the PDA or had no preference. PDA found easy to use	Power – 0/1 (Total – 21/27)
						Age did not correlate with difficulty using PDA	
Sandgren et al., 2003 North Dakota, USA [19]	222 women with breast cancer	6×30 min telephone therapy sessions that involved either cancer education or emotional expressions	Standard care	5 months	Perceived control	Cancer education group reported greater perceived control compared to standard care	Study Quality – 7/10
					Mood		
					Quality of life	No difference for mood or quality of life	External validity – 2/3
							Internal Validity (bias) – 5/7
							Internal Validity (selection bias) – 6/6
							Power – 1/1 (Total – 21/27)
Sikorski et al., 2009 Michigan, USA [22]	486 cancer patients	Automated voice response symptom reporting	Nurse assisted symptom management via the telephone	6 telephone contacts over 8 weeks	Severity of cancer symptom at intake interview and at first intervention contact	Patient in the AVR group reported more severe symptoms. There was a variation with age with older patients reporting more severity of symptoms to the nurse	Study Quality – 9/10
							External validity - 2/3
							Internal Validity (bias) – 5/7
							Internal Validity (selection bias) – 6/6
							Power – 0/1 (Total – 22/27)
Sikorskii et al., 2007 Michigan, USA [23]	435 cancer patients	Automated telephone symptom management	Nurse-assisted symptom management	10 weeks	Severity of cancer symptoms, demographic data and co-morbidities	Reduction in symptom severity in both groups. Lung cancer patients with greater symptom severity withdrew from the ATSM group	Study Quality – 8/10
							External validity – 2/3
							Internal Validity (bias) – 5/7
							Internal Validity (selection bias) – 6/6
							Power – 1/1 (Total – 22/27)
Yun et al. 2012 Seoul, Korea [24]	273 cancer patients	Internet based, individually tailored cancer related fatigue education program	Usual care	12 weeks	Level of fatigue	Education group reported a reduction in fatigue, decrease in HADS anxiety score, increase in global QoL score and emotional, cognitive and social functioning of EORTIC QLQ-C30	Study Quality – 8/10
					Quality of Life, Anxiety and depression		External validity – 1/3
							Internal Validity (bias) – 4/7
							Internal Validity (selection bias) – 6/6
							Power – 1/1 (Total – 20/27)

**Table 2:** Details of intervention, outcomes, measures used and results

**Study**	**Intervention**	**Primary and secondary outcomes**	**Measures used**	**Results including statistical values**
Beaver et al. (2009) [11,25]	Telephone follow up by specialist nurses	Psychological Morbidity	State-trait anxietntdy inventory	No difference for psychological morbidity
		Participant’s need for information	General Health Questionnaire	Patients in telephone group more satisfied (intention to treat p < 0.001)
		Participants’ satisfaction		No difference for information needs
		Clinical investigations ordered		No difference for clinical investigations
		Time to detection of recurrent disease		Recurrences :- no differences between the two groups p = 0.295% CI (−3.33-2.07) – equivalence demonstrated. 28)
Davison et al. (2002) [15]	Computer programme providing information and assisting decision making	“Extent to which women achieved their preferred decisional roles”	Control Preferences Scale (CPS)	Intervention group was more passive in decision making than planned. (p < 0.0001)
		Patient satisfaction	Patient Satisfaction Questionnaire (PSQ)	More women over 50 opted to play a more passive role. (p= > 0.002)
				No difference found for the two groups for patient satisfaction. Both groups reported high levels
Harrison et al. (2011) [21]	5 telephone calls from a specialist colorectal nurse in 6 months after discharge	Unmet supportive care needs	SCNS-SF34 and FACT-C used for unmet supportive care needs and quality of life. CaSUN was used to measure these two outcomes at 6 months	No difference was found for unmet supportive care needs at 6 months
		Secondary outcomes:	Patient asked to remember health service use in a telephone interview	“Observed effect size for supportive care needs was 0.25“
		Health service utilization		Study was aiming for effect size of 0.75.
		Quality of life		Quality of life had improved by “twice as much” in the intervention group at six months. (size of difference = 5.7)
				At 6 months in the intervention group; fewer “presentations to emergency departments (p = 0.23) and readmissions to hospital (p = 0.37)” compared with the control group. Intervention group patients had more contact with “hospital-based, specialist based and community services”. Differences for health service utilization were not statistically significant
Hegel et al. (2010) [16]	6 weekly session of telephone delivered problem solving occupational therapy	Feasibility of conducting a RCT including patient satisfaction	Study recruitment and retention data was gathered	“67% recruitment rate (31/46)”
		**Secondary outcomes:**	At 12 weeks participants completed a satisfaction survey	“81% retention rate”
		**Functional quality of life**		“92% of those receiving the intervention were “highly satisfied”
		**Emotional status**		92% reported it to be “helpful/very helpful”
				97% if planned sessions of the intervention were completed
				Effect sizes were calculated for secondary outcomes but “study was not powered to detect treatment effects”. Main outcome was feasibility for study to be repeated as larger scale RCT. No CIs quoted
Kearney et al. (2008) [12]	Mobile phone-based remote monitoring during chemotherapy	“Chemotherapy-related morbidity” of six symptoms	Electronic symptom questionnaire completed by patients in control and intervention group before the start of chemotherapy and prior to cycles 2, 3, 4 and 5	In the control group more report of fatigue (CI = 1.04-5.05, P = 0.040) and lower reporting of hand-foot syndrome (CI 0.17-0.92 P = 0.031) Severity and distress of symptoms were no different between the two groups except for hand-foot syndrome in the intervention group. (Severity CI −0.52 to −0.02 P = 0.033, Distress CI −0.33 to −0.02, P = 0.028). Other differences were not statistically significant
		These were nausea, vomiting, fatigue, mucositis, hand-foot syndrome and diarrhoea”		
Kimman et al., 2011 [17,27]	Nurse led telephone follow up or	Health-related quality of life (HRQoL)	HRQoL: EORTC QLQ-C30	No difference between the two groups for HRQoL. (P value = 0.42
	Nurse led telephone follow up plus educational group programme (EGP)	Secondary outcomes:	Role and emotional functioning: EORTC QLQ-C30 subscales	Confidence interval of 95% for the “estimated difference between mean HRQoL scores at 12 months after treatment” = −1.93-4.64)
		Role and emotional functioning	Anxiety: State Trait Anxiety Inventory (STAI)	No differences between groups for all other secondary outcomes (all p values > 0.05)
		Feelings of control and anxiety	Perceived feelings of control: Mastery scale	
Kroenke et al. (2010) [26]	Centralized telecare management by a nurse-physican specialist team coupled with home-based symptom monitoring by interactive voice recording or internet	Depression and pain	Measured at baseline and at 1, 3, 6 and 12 months. Depression measured using the “20-item Hopkins Symptom Checklist (HSCL-20) and pain (BPI) severity	Greater improvements in pain (p < 0.001) and depression (p < 0.001) in the intervention group.
		Secondary outcomes:	Health related quality of life: SF-12	Effect size “for between-group differences” at 12 months for pain were 0.39 (95% CI, 0.01-0.77) and for depression, 0.41 (95% CI, 0.08-0.72)
		Health-related quality of life	“Quality of life – single item 0-10”	No difference for health-related quality of life and health-care use. Difference for “other pain treatments” (p = 0.03).
		Disability	Anxiety – “7-item Generalised Anxiety Disorder scale”	
		Cointerventions	“Physical symptom burden – 22-item somatic symptom scale”	
		Self reported health care use	Fatigue- “SF 36 vitality scale”	
			Disability – “3- item Sheehan Disability Scale”	
			“Self-report health care use: treatment survey”	
Marcus et al. (2009) [18]	16 session telephone counselling post treatment	Cancer specific Distress	Cancer specific Distress – Impact of Event Scale (IES).	No differences found for depression and distress unless end points were “dichotomized at cutpoints suggestive of the need for clinical referral”. A 50% reduction in depression and distress was demonstrated in the intervention group compared to the control. (p = 0.07)
		Depression	Depression – Centre for Epidermiologic Studies Depression Scale (CES-D)	“Significant effects” shown in sexual dysfunction and personal growth for the intervention group
		Sexual dysfunction	Sexual Dysfunction – 25 items (designed for study)	When endpoints dichotomized- no change in the control group (depression: p = 0.41, distress = 0.86). Intervention group (depression p = 0.0007, distress p = 0.0007)
		Personal Growth	Personal growth – 5 items (designed for study)	Group differences at 18 months were significant
				Depression: p = 0.06 and Distress: p = 0.07 “with effect sizes of 0.23 and 0.24”
				Sexual dysfunction: at 18 months, “significant improvement intervention group” p = 0.04, effect size = 0.23
				Personal growth- both groups improved but more in the intervention group. (At 18 months p = 0.03, effect size =0.22)
Matthew et al. (2007) [20]	PDA survey followed by paper	Data quality	International Prostate Symptom Score (IPSS)	Internal consistency found to be high
	PDA followed by PDA survey. (3 groups)	Feasibility	Patient Orientated Prostate cancer Utility Survey (PORPUS) International Index of Erectile Function-5 (IIEF-5) either in paper or PDA forms	Test re-test reliability high. (p < 0.01)
				Scores across modalities were correlated demonstrating “concurrent validity (p < 0.01)”
				No differences in levels of participation
				Preference was highest for the PDA version of the questionnaire. (58.6%)
				Age did not have an impact on preference (p = 0.12)
				Age did not have an impact of difficulty using PDA. (p = 0.08)
				Confidence intervals quoted in the paper for each of the data items within the questionnaire
Sandgren et al. (2003) [19]	6×30 min telephone therapy sessions that involved either cancer education or emotional expressions	Mood	Quality of Life – Functional Assessment of Cancer Therapy-Breast Instrument (FACT-B)	Cancer Education group – greater perceived control (p < 0.01)
		Quality of Life	Mood – Profile of Mood States	No difference for mood (p > 0.12) or quality of life (p > 0.12) found
		Perceived control	Perceived control – Perceived Stress Scale	No CIs quoted- only standard deviations, means and p values
Sikorskii et al. (2007) [22]	Automated telephone symptom management	Severity of symptoms	17 symptoms scored for severity – designed for the study. Analysed using a RASCH model	Both groups had a reduction in symptom severity. No difference found between 2 groups
				Effect sizes were almost the same for NASM (0.56) and ATSM (0.59)
Sikorskii et al. (2009) [23]	Automated voice response symptom reporting	Severity of symptoms – difference depending on mode of assessment	14 cancer related symptoms – scored for severity. Designed for the study	AVR group reported more severe symptoms of “nausea, vomiting, diarrhoea, poor appetite, pain and alopecia (p values less than 0.05)”
				Varied with age with older patients reporting higher severity in the nurse led group (effect sizes greater or equal to 0.35”)
Yun et al. 2012 Seoul, Korea [24]	Internet based, individually tailored cancer related fatigue education program	Level of fatigue	Brief Fatigue Inventory (BFI)	Intervention group reported an improvement in fatigue with a significantly greater decrease in BFI global score (−0.66 points, 95% CI −1.04 to −0.27), FSS total score (−0.49;95% CI, 0.78 to −0.21) and HADS score
		Anxiety and depression	Fatigue Severity Scale (FSS)	Participants with moderate or greater fatigue reported a significantly greater decrease in HADS Anxiety score (−0.90; 95%CI, −1.51 to −0.29) as well as global quality of life (5.22; 95% CI, 0.93 to 9.50) and several functioning scores of the EORTC QLQ_C30
		Global quality of life	Hospital Anxiety and Depression Scale (HADS)	
			European Organisation for Research and Treatment of Cancer Quality of Life Questionnaire C30 (EORTC QLQ_C30)	

#### Included patients and countries

Of the thirteen included studies eight had been conducted amongst women with breast cancer; one amongst men with prostate cancer and one amongst people with colorectal cancer [11,13,15-21]. Of the remaining three, a Scottish study using mobile phones to monitor symptoms was conducted in patients with breast, lung or colorectal cancer currently undergoing chemotherapy, a US study included patients with any solid tumour or non-Hodgkin’s lymphoma who were currently undergoing chemotherapy and a Korean study of cancer survivors who had reported severe fatigue [12,22-24]. One study was conducted in the Netherlands [17], one in Australia [21], two each in the UK [11,12,25], and Canada [15,20], six in the USA [16,18,19,22,23,26] and one in Korea [24]. The age ranges of patients were not consistently recorded.

### Synthesis of key outcomes

#### Range of interventions and technologies employed

Of the 13 randomised studies the majority, seven, of the interventions were relatively low-tech and had simply employed standard telephone calls to cancer aftercare recipients in their own homes, as an alternative to standard follow-up [11,13,16-19,21]. These calls were generally delivered by specialist nurses. The content, duration and frequency varied across studies. Calls were mainly scheduled, regular, and lasted approximately 30 minutes, and delivered components of symptom monitoring, information sharing, and emotional support. Two further interventions employed remote symptom monitoring using a smartphone/personal digital assistant (PDA) [12,20]. A further intervention employed an automated voice activated telephone response system to monitor symptom severity [22,23]. One intervention comprised a computer programme, completed by those in aftercare, which provided the patient with information and assisted decision making [15]. Participants were encouraged to work through the computer programme prior to their face to face follow-up meeting, the aim being to enable them to participate more fully in their subsequent follow-up consultation [15]. One study sought to combine technologies in a centralised telephone management system operated by a specialist nurse [26]. Patients’ symptoms were monitored in their own home using an internet based application and/or a voice activated telephone helpline with nurses telephoning patients back if symptoms indicated this was required. The final study developed Health Navigation, an Internet-based individually tailored education programme for patients suffering from cancer related fatigue. Participants had access to the Health Navigation website and were guided through a twelve week programme including sessions on energy conservation, physical activity, nutrition, sleep hygiene, pain control and distress management [24].

#### Patient acceptability/satisfaction

Data on patient satisfaction or acceptability was explicitly reported in five of the trials [11,13,15,16,25]. Beaver et al. reported that most women with breast cancer had equivalent satisfaction with elements of cancer follow-up received via technological means compared to usual care [11,25]. Kimman et al. found that nurse-led telephone follow-up after curative treatment for breast cancer resulted in high satisfaction scores with the added potential to reduce clinic visits [13]. In the study of symptom monitoring using paper or PDA surveys in men with prostate cancer, most preferred using the PDA and found it easy to use, and older age did not appear to be associated with reports of difficulty in using the PDA [15]. A smaller feasibility trial, reported that 92% of US women with breast cancer who received 6 weeks of telephone delivered problem solving occupational therapy were highly satisfied with the intervention [16]. There was no evidence from the other studies that technological interventions led to reduced patient satisfaction with follow-up care.

#### Clinical safety

Only one study reported on clinical safety [11,25]. It was conducted amongst 374 women with breast cancer and reported on time to detection of recurrence, with no significant differences observed between intervention and control groups.

#### Health-related quality of life

In a Scottish study of remote monitoring of symptoms in patients undergoing chemotherapy intervention group patients were less fatigued and reported more hand and foot syndrome [12]. Similarly, in a US trial of chemotherapy related symptom monitoring patients receiving symptom monitoring via an automated telephone symptom monitoring system reported more severe symptoms than those receiving nurse assisted symptom management [22,23]. After 10 weeks symptom severity, compared to baseline, had reduced in both groups [23]. An Australian study of patients with colorectal cancer receiving telephone follow-up from a specialist nurse, quality of life scores were higher in the intervention group at six months [21]. Intervention participants in the Korean fatigue study [24] reported a decrease in 12 week scores compared to baseline in both the Brief Fatigues Inventory and Fatigue Severity Scale as well as a reduction in anxiety and several functioning scores of the EORTC-C30. However a study of 299 breast cancer patients in the Netherlands reported no significant difference in the health-related quality of life between the intervention group receiving telephone follow up and the control group receiving hospital follow up [17]. Taking these six trials together there was no evidence of significantly increased psychological distress or reduced quality of life in any of the intervention groups.

#### Health economic outcomes

Only two studies included any assessment of health economic outcomes with only one study reporting specifically on cost [25]. Beaver et al. conducted a cost minimisation analysis of specialist nurse-led follow-up [25]. This concluded that telephone follow-up was more costly for health services, with patients receiving telephone follow–up having approximately 20% more consultations than those receiving hospital follow-up, in addition telephone consultations were longer than clinic consultations and requests for mammography or additional referrals were greater in this group, however telephone follow-up was less costly for patients [25]. It should be noted that many of the health service costs related to training to deliver the intervention, costs which would subsequently decline [25]. On the other hand, Dutch investigators, Kimman et al., [27] concluded that standard hospital follow-up plus an educational group programme resulted in greater gain in QALYs compared to telephone follow-up, but at considerable cost possibly due to the high level of contacts made by the telephone follow-up group with specialised healthcare professionals and their costs of lost production [27]. In a subgroup analysis included within this study hospital follow-up and an educational group programme was found to be maximally cost effective in anxious patients. Age, level of education, and chemotherapy did not influence cost-effectiveness [27]. Beaver et al. did not conduct a sub-group analysis so comparisons could not be made at this level [25].

In the other studies, whilst not formally reporting any health economic evaluation, there was no evidence of increased health care utilisation amongst recipients of the intervention in fact one noted a significant reduction in subsequent referrals for depression and distress amongst 304 US breast cancer patients that had received telephone follow-up [18].

### Critical appraisal of studies

A standard checklist was used to critically appraise the included studies using standard criteria [14]. Scores are shown in Table 1 and ranged from 17 to 23. One study attracted a quality score of 6/10, mainly due to poor expression of the study aims and description of the participants [15]. Thus, these studies were generally of good quality and reported to a high standard. In all cases the researchers had addressed issues of internal validity and recognised the issues created by not being able, in most, cases to blind participants to the intervention. Most studies failed to employ blinded data collection. In all studies there was good recognition of, and controlling of, potential confounding leading to high scores in this domain. Most of the studies were, however, underpowered with respect to clinical outcomes. The main issue of quality with the study was a failure to demonstrate external validity. Only four of the included studies included information on non-respondents, generally reporting that they were older and of lower educational status [11,17,18,20]. This underscores the fact that technology trials as currently reported exhibit considerable recruitment bias. This was further illustrated by some of the studies not reporting on non-respondents acknowledging that this was a limitation and highlighting the fact that they had, for example, recruited only urban or white participants [15,18,28].

## Discussion

### Summary of evidence

The limited evidence available suggests that using technology for cancer follow-up is acceptable to patients and clinically safe. However, there is currently insufficient evidence to definitively state whether or not remote cancer follow-up using technology is likely to be cost effective, since most existing randomised trial evidence relates to alternative models of follow-up using standard telephone calls only. The results are important as a prompt and guide to future research since, in the face an ageing population and increasing number of cancer survivors, the need for safe, acceptable and economically viable alternatives to current resource intensive cancer follow-up care delivery models is urgent. Existing evidence suggests that scheduled cancer follow-up can employ elements of technology without reducing patient satisfaction, compromising safety, impairing quality of life or increasing psychological distress.

### Strengths and limitations

As far as we aware this is the first systematic review to focus on the use of technology to deliver cancer follow-up. The methods of the review were thorough and robust and the authors have confidence that the search strategy was suitably inclusive to identify most relevant studies. We chose to include only randomised controlled trials in the review and did not include other studies types, such as interrupted time series studies. This was principally due to our concern that trials of new technologies are particularly prone to selection bias (i.e. the recruitment of enthusiasts). Whilst the randomised trial is not a panacea to this problem in technological research we believe that by restricting our review to a method which explicitly addresses this issue was the best approach. A further issue is that the CONSORT statement which promotes quality in the reporting has been widely accepted and implemented. Furthermore randomised trials, in general, include harder outcome. We believe our results have borne out our approach.

The review was limited to studies written in English. Thus it is possible that some studies conducted in other parts of the world were missed. This is particularly so when it is considered that many non-English speaking countries, for example, Germany are key-players in technological innovation. We do think, however, that since English is practically the “lingua franca” of medical research it is unlikely that we have missed any major randomised trial by employing this limitation. It does seem most likely that these would have been published in a journal employing the English language for which the search strategy was exhaustive. Further support for this view can be obtained from the fact that both trials currently registered on the ISRCTN register of using technology in cancer follow-up are in non-English speaking countries but fully described and registered in English.

The majority of existing studies were conducted in the developed world in women with breast cancer, a group which will potentially represent the more enabled and affluent end of the cancer survivorship spectrum. This limits the extension of the current findings to other settings, cancers and patient groups, emphasising the need for further research. It could also be that there are patient groups in whom it would be inappropriate to attempt to utilise technology in their follow-up, an issue that the current evidence goes no way toward identifying or addressing. Furthermore, there was insufficiently consistent reporting within the studies of inclusion criteria with respect to time since completion of treatment or stage at diagnosis to draw meaningful conclusions with respect to these parameters. A further limitation has occurred in the synthesis and interpretation of the evidence. There were surprisingly few randomised studies identified and the majority used telephone follow-up only, with only a few adopting more recent technological innovations such as PDAs. Thus, given the increasing potential of modern digital technologies the included interventions were relatively simple and low-tech. Furthermore, the way in which technology could enable patients to send information to healthcare professionals has not yet been meaningfully explored. Although the interventions addressed several aspects of follow-up care (recurrence monitoring, monitoring treatment effect, information provision) none were comprehensive, and most used technology to deliver one aspect of follow-up care as an adjunct to more traditional follow-up models. Because of this direct comparison of interventions was extremely difficult given the different technologies, interventions and outcomes reported. Nevertheless, we believe the current review provides good evidence of the potential of technology to deliver safe effective cancer follow-up in the future. The review also highlights the need for future trials to seek to provide definitive information on patient acceptability, clinical safety and cost effectiveness.

### Context with other literature

There is a growing recognition that cancer follow-up services are increasingly stretched by a growing population of cancer survivors [1,3,6]. This has prompted researchers to develop alternative models of care which have utilised different healthcare professionals in care delivery and/or shifted the focus of care from hospital to the community [4,29,30]. Despite, this, these models will probably continue to consume a large and growing amount of healthcare resource and still inconvenience certain patient groups. The randomised studies included within this review are striking in the respect that they demonstrate that few mature complex interventions utilising technology, other than standard telephone calls, have been developed to a standard sufficient for subjection to a randomised controlled trial. The need to do this is, perhaps, one of the key implications of our work. There is an emerging vision that modern technologies, evidenced by policy initiative worldwide, could potentially enhance current models or offer other alternatives to the future delivery of high quality cancer care [31-38]. There is considerable evidence from other disease areas of using modern technologies to good effect [39]. For example, text messaging has been found to increase self-efficacy and adherence to medication in young people with diabetes and has been proven to be an effective aid to smoking cessation [40,41]. Online systems which enable patients to access their own health records have enjoyed some success as a means to educate patients and assist in their self-management, for example, in the USA, Kaiser Permanente’s My Health Manager has demonstrated significant reductions in primary care contacts [42]. The CHAMPION project has enabled disabled adults to improve communication with healthcare professionals by inputting information on their own care needs into a database, for example, uploading a video to demonstrate to clinical staff how they use their communication devices [43]. However, there is now a striking need to develop high quality and innovative technological interventions to support growing numbers of people with cancer.

Inspection of the International Standard Randomised Controlled Trial Number Register indicates that increasing numbers of clinical trials of technological applications to healthcare are occurring [44]. The register lists twenty-three active trials on telehealth, telemedicine or ehealth, including trials in men with prostate cancer and another amongst patients with breast or colorectal cancer [45,46]. Nevertheless, this is surprisingly few randomised trials given the explosion in technological innovation in recent years. It could be that technology is evolving so fast that potential innovative technological interventions become outdated before they can mature sufficiently to be subjected to randomised trials.

## Conclusions

At the current time there are few well conducted randomised trials exploring the role of digital and other technologies in the delivery of structured cancer aftercare. There is, however, some evidence that modern technologies can be used to safely and effectively deliver aspects of structured cancer follow-up as an alternative or adjunct to existing traditional models which require patients to travel to tertiary cancer centres for face to face visits with a cancer specialist. Given the potential gains offered by digital technology in terms of patient convenience and empowerment and reduction of resource use in cancer centres, there is an urgent need for future cancer research to embrace the digital age and seek to integrate various different technologies into innovative and comprehensive models of virtual cancer follow-up. It is essential, however, that such trials employ robust and consistent measures of patient satisfaction and acceptability, clinical safety and cost effectiveness so that powerful evidence on these outcomes can accumulate.

## Competing interests

All authors have seen and approved the final manuscript. They do not have any competing interests to declare. The authors have full control of all primary data and they agree to allow the journal to review their data if requested.

## Authors’ contributions

RD, CB and PM designed the review. RD conducted the review assisted by SH. JS participated in updating the review. RD and PM wrote the manuscript with comments on drafts from SH, JS and CB. All authors read and approved the final manuscript.

## Pre-publication history

The pre-publication history for this paper can be accessed here:

http://www.biomedcentral.com/1471-2407/14/311/prepub

## Supplementary Material

Additional file 1 Search strategy used for embase. Click here for file

## References

[B1] Brennan ME Butow P Marven M Spillane AJ Boyle FM Survivorship care after breast cancer treatment- Experiences and preferences of Australian women Breast 2011 20 271 277 10.1016/j.breast.2010.12.006 21236671

[B2] Macbride SK Whyte F Survivorship and the cancer follow-up clinic Eur J Cancer Care 1998 7 47 55 10.1046/j.1365-2354.1998.00065.x 9582751

[B3] Hall S Samuel L Murchie P Shared follow-up for cancer: developing the model with patients and GPs Fam Pract 2011 28 554 564 10.1093/fampra/cmr012 21467132

[B4] Lewis R Neal R Williams N France B Hendry M Russell D Hughes D Russell I Stuart N Weller D Wilkinson C Follow up of cancer in primary care versus secondary care: a systematic review Br J Gen Pract 2009 59 e234 e247 19566990 10.3399/bjgp09X453567 PMC2702037

[B5] Maddams J Brewster D Gavin A Steward J Elliot J Utley M Moller H Cancer prevalence in the United Kingdom: estimates for 2008 Br J Cancer 2009 101 541 547 10.1038/sj.bjc.6605148 19568236 PMC2720244

[B6] Okera M Baker NA Hayward AM Selva-Nayagam S Oncology workforce issues: the challenge of the outpatient clinic Intern Med J 2011 41 499 503 10.1111/j.1445-5994.2011.02506.x 21707896

[B7] Green J Murchie P Lee AJ Does place of residence affect the management of cutaneous melanoma? Analysis of a database from Northern Scotland J Rural Health 2013 doi: 10.1111/jrh.12011 10.1111/jrh.12011 23944278

[B8] NHS Improvement Rapid Review of current service provision following cancer treatment Available at http://www.ncsi.org.uk/ (accessed 15th May 2013)

[B9] Sood S Mbarika V Jugoo S Dookhy R Doarn CR Prakash N Merrell RC What is telemedicine? A collection of 104 peer-reviewed perspectives and theoretical underpinnings Telemed J E Health 2007 13 573 590 10.1089/tmj.2006.0073 17999619

[B10] McLean S Protti D Sheikh A Telehealthcare for long term conditions BMJ 2011 342 374 378 10.1136/bmj.d374 21292710

[B11] Beaver K Tysver-Robinson D Campbell M Twomey M Williamson S Hindley A Susnerwala S Dunn G Luker K Comparing hospital and telephone follow-up after treatment for breast cancer: randomised equivalence trial BMJ 2009 338 a3147 10.1136/bmj.a3147 19147478 PMC2628299

[B12] Kearney N McCann L Norrie J Taylor L Gray P McGee-Lennon M Sage M Miller M Maguire R Evaluation of a mobile phone-based, advanced symptom management system (ASyMS) in the management of chemotherapy-related toxicity Support Care Cancer 2009 17 437 444 10.1007/s00520-008-0515-0 18953579

[B13] Kimman ML Bloebaum MMF Dirksen CD Houben RMA Lambin P Boersma LJ Patient satisfaction with nurse-led telephone follow-up after curative treatment for breast cancer BMC Cancer 2010 10 174 10.1186/1471-2407-10-174 20429948 PMC2880988

[B14] Downs S Black N The feasibility of creating a check-list for the assessment of the methodological quality both of randomised and non-randomised studies of health care interventions J Epidemiol Community Health 1998 52 377 384 10.1136/jech.52.6.377 9764259 PMC1756728

[B15] Davison BJ Degner LF Feasibility of using a computer-assisted intervention to enhance the way women with breast cancer communicate with their physicians Cancer Nurs 2002 25 417 424 10.1097/00002820-200212000-00001 12464832

[B16] Hegel MT Lyons KD Hull JG Kaufman P Urquhart L Li Z Ahles TA Feasibility study of a randomized controlled trial of a telephone-delivered problem-solving-occupational therapy intervention to reduce participation restrictions in rural breast cancer survivors undergoing chemotherapy Psychooncology 2011 20 1092 1101 10.1002/pon.1830 20821373 PMC3005985

[B17] Kimman ML Dirksen CD Voogd AC Falger P Gijsen BCM Thuring M Lenssen A Van Der Ent F Verkeyn J Haekens C Hupperets P Nuytinck JKS Van Riet Y Brenninkmeijer SJ Scheijmans LJEE Kessels A Lambin P Boersma L 2011. Nurse-led telephone follow-up and an educational group programme after breast cancer treatment: Results of a 2×2 randomised controlled trial Eur J Cancer 2011 47 1027 1036 10.1016/j.ejca.2010.12.003 21237636

[B18] Marcus AC Garrett KM Cella D Wenzel L Brady MJ Fairclough D Pate-Willig M Barnes D Emsbo SP Kluhsman BC Crane L Sedlacek S Flynn PJ Can telephone counselling post-treatment improve psychosocial outcomes among early stage breast cancer survivors? Psychooncology 2010 19 923 932 10.1002/pon.1653 19941285 PMC2891611

[B19] Sandgren AK McCaul KD Short-term effects of telephone therapy for breast cancer patients Health Psychol 2003 22 310 315 12790259 10.1037/0278-6133.22.3.310

[B20] Matthew AG Currie KL Irvine J Ritvo P Santa Mina D Jamnicky L Nam R Trachtenberg J Serial personal digital assistant data capture of health-related quality of life: a randomized controlled trial in a prostate cancer clinic Health Qual Life Outcomes 2007 5 38 10.1186/1477-7525-5-38 17617906 PMC1936985

[B21] Harrison JD Young JM Solomon MJ Butow PN Secomb R Masya L Randomized pilot evaluation of the supportive care intervention “CONNECT” for people following surgery for colorectal cancer Dis Colon Rectum 2011 54 622 631 10.1007/DCR.0b013e31820bc152 21471765

[B22] Sikorskii A Given CW Given B Jeon S You M Differential symptom reporting by mode of administration of the assessment: automated voice response system versus a live telephone interview Med Care 2009 47 866 874 10.1097/MLR.0b013e3181a31d00 19584761 PMC2722377

[B23] Sikorskii A Given CW Given B Jeon S Decker V Decker D Champion V McCorkle R Symptom management for cancer patients: a trial comparing two multimodal interventions J Pain Symptom Manage 2007 34 253 264 10.1016/j.jpainsymman.2006.11.018 17618080 PMC2043403

[B24] Yun YH Lee KS Kim KW Park SY Lee ES Noh DY Kim S Oh JH Jung SY Chung KW Lee YJ Jeong SY Park KJ Shim YM Zo JI Park JW Kim YA Shon EJ Park S Web-based tailored education program for disease-free cancer survivors with cancer related fatigue: A randomised controlled trial J Clin Oncol 2012 30 1296 1303 10.1200/JCO.2011.37.2979 22412149

[B25] Beaver K Hollingworth W McDonald R Dunn G Tysver-Robinson D Thomson L Hindley AC Susnerwala SS Luker K Economic evaluation of a randomized clinical trial of hospital versus telephone follow-up after treatment for breast cancer Br J Surg 2009 96 1406 1415 10.1002/bjs.6753 19918858

[B26] Kroenke K Theobald D Wu J Norton K Morrison G Carpenter J Tu W Effect of telecare management on pain and depression in patients with cancer: a randomized trial JAMA 2010 304 163 171 10.1001/jama.2010.944 20628129 PMC3010214

[B27] Kimman ML Dirksen CD Voogd AC Falger P Gijsen BC Thuring M Lenssen A van der Ent F Verkeyn J Haekens C Hupperets P Nuytinck JK van Riet Y Brenninkmeijer SJ Scheijmans LJ Kessels A Lambin P Boersma LJ Economic evaluation of four follow-up strategies after curative treatment for breast cancer: results of an RCT Eur J Cancer 2011 47 1175 1185 10.1016/j.ejca.2010.12.017 21257305

[B28] Van den Brink JL Moorman PW de Boer M Hop WCJ Pruyn JFA Verwoerd CDA van Bemmel JH Impact on quality of life of a telemedicine system supporting head and neck cancer patients: a controlled trial during the postoperative period at home J Am Med Inform Assoc 2006 14 198 205 17213498 10.1197/jamia.M2199 PMC2213461

[B29] Cox K Wilson E Follow-up for people with cancer: nurse-led services and telephone interventions J Adv Nurs 2003 43 51 61 10.1046/j.1365-2648.2003.02672.x 12801396

[B30] Murchie P Nicolson MC Hannaford PC Raja EA Lee AJ Campbell NC Patient satisfaction with GP-led melanoma follow up: a randomised controlled trial Br J Cancer 2010 102 1447 1455 10.1038/sj.bjc.6605638 20461089 PMC2869159

[B31] Hazin R Qaddoumi I Teleoncology: current and future applications for improving cancer care globally Lancet Oncol 2010 11 204 210 10.1016/S1470-2045(09)70288-8 20152772 PMC3157842

[B32] Hede K Teleoncology gaining acceptance with physicians, patients J Natl Cancer Inst 2010 102 1531 1533 10.1093/jnci/djq426 20935264

[B33] Palkhivala A Canada develops models of teleoncology J Natl Cancer Inst 2011 103 1566 1567 10.1093/jnci/djr449 22010180

[B34] Sudhamony S Nandakumar K Binu PJ Issac NS Telemedicine and tele-health services for cancer-care delivery in India IET Commun 2008 2 231 236 10.1049/iet-com:20060701

[B35] Allen A Hayes J Patient satisfaction with teleoncology: a pilot study Telemed J 1995 1 41 46 10.1089/tmj.1.1995.1.41 10165321

[B36] Ricke J Bartelink H Telemedicine and its impact on cancer management Eur J Cancer 2000 36 826 833 10.1016/S0959-8049(00)00057-5 10785586

[B37] Coelho JJ Arnold A Nayler J Tischkowitz M MacKay J An assessment of the efficacy of cancer genetic counselling using real-time videoconferencing technology (telemedicine) compared to face-to-face consultations Eur J Cancer 2005 41 2257 2261 10.1016/j.ejca.2005.06.020 16176873

[B38] Weinstein RS López AM Barker GP Krupinski EA Descour MR Scott KM Richter LC Beinar SJ Holcoms MJ Bartels PH McNeely RA Bhattacharyya AK The innovative bundling of teleradiology, telepathology, and teleoncology services IBM Syst J 2007 46 69 84

[B39] Ekeland AG Bowes A Flottrop S Effectiveness of telemedicine: a systematic review of reviews Int J Med Inform 2010 79 736 771 10.1016/j.ijmedinf.2010.08.006 20884286

[B40] Franklin VL1 Waller A Pagliari C Greene SA A randomized controlled trial of Sweet Talk, a text-messaging system to support young people with diabetes Diabet Med 2006 23 1332 1338 10.1111/j.1464-5491.2006.01989.x 17116184

[B41] Bennett DA Emberson JR Text messaging in smoking cessation: the txt2stop trial Lancet 2011 378 6 7 10.1016/S0140-6736(11)60882-9 21722951

[B42] Kaiser Permanente (2012) My health manager 2012 https://healthy.kaiserpermanente.org/health/care/consumer/my-health-manager. (Accessed 20 Feb 2014)

[B43] Prior S Involving adults with severe speech and physical impairments in the design of CHAMPION ACM SIGCHI conference on human factors in computing systems 2010 Atlanta, USA: ACM

[B44] International Standard Randomised Controlled Trial Number Register 2014 http://www.controlled-trials.com/isrctn/search.html (accessed 20th February 2014)

[B45] Eysenbach G Impact of Internet Instructions on Men with Prostate Cancer ISRCTN10001875. doi:10.1186/ISRCTN10001875. http://www.controlled-trials.com/isrctn/search.html (accessed 20th February 2014)

[B46] Orruño E Evaluation of an e-health intervention for cancer patients’ support ISRCTN00735390. doi:10.1186/ISRCTN00735390. http://www.controlled-trials.com/isrctn/search.html (accessed 20th February 2014)

