# Supplementary material for: Using technology to deliver cancer follow-up: a systematic review
Source: BMC Cancer. 2014 May 3;14:311. doi: 10.1186/1471-2407-14-311 (PMC4101828; doi:10.1186/1471-2407-14-311)
Supplement: Additional file 1 — Search strategy used for embase. [file 1471-2407-14-311-S1.doc]

**Additional file 1: Search Strategy used for Embase.**

**Embase 1996-2011 (8^th^ February 2014)**

1. exp neoplasm/

2. cancer.tw.

3. exp follow up/

4. follow?up.tw.

5. exp aftercare/

6. after?care.tw.

7. exp telemedicine/

8. tele?medicine.tw.

9. exp medical society/ or exp decubitus/ or exp United States/ or exp in service training/ or exp telecommunication/ or exp legal aspect/ or exp health care policy/ or exp telemedicine/ or exp telehealth/ or exp Internet/ or exp health service/

10. tele?health$.tw.

11. exp error/ or exp lung transplantation/ or exp female infertility/ or exp mouse/ or exp society/ or exp spike/ or exp amyloidosis/ or exp heart/ or exp embryonic stem cell/ or exp oral contraceptive agent/

12. technolog$.tw.

13. exp gene technology/ or exp process technology/ or exp dental technology/ or exp "genetic engineering and gene technology"/ or exp medical technology/ or exp biomedical technology assessment/ or exp recombinant DNA technology/ or exp technology/ or exp information technology/ or exp assistive technology device/ or exp educational technology/ or exp assistive technology/ or exp membrane technology/ or exp surgical technology/ or exp "engineering and technology"/

14. exp gene technology/ or exp process technology/ or exp dental technology/ or exp "genetic engineering and gene technology"/ or exp medical technology/ or exp biomedical technology assessment/ or exp recombinant DNA technology/ or exp technology/ or exp information technology/ or exp assistive technology device/ or exp educational technology/ or exp assistive technology/ or exp membrane technology/ or exp surgical technology/ or exp "engineering and technology"/

15. technology.tw.

16. technologies.tw.

17. exp telecommunication/

18. tele?communication$.tw.

19. 1 or 2

20. 3 or 4

21. 5 or 6

22. 20 or 21

23. 7 or 8

24. 9 or 10

25. 13 or 14 or 15 or 16

26. 17 or 18

27. 11 or 12

28. 19 and 22 and 23

29. limit 28 to (english language and yr="2000 - 2011")

30. 19 and 22 and 24

31. limit 30 to (english language and yr="2000 - 2011")

32. 19 and 22 and 25

33. limit 32 to (english language and yr="2000 - 2011")

34. 19 and 22 and 26

35. limit 34 to (english language and yr="2000 - 2011")

36. 19 and 22 and 27

37. limit 36 to (english language and yr="2000 - 2011")

38. exp telemedicine/ or exp telehealth/ or exp Internet/ or exp telecommunication/

39. 10 or 38

40. 19 and 22 and 39

41. limit 40 to (english language and yr="2000 - 2011")

42. exp telemedicine/ or exp health care policy/ or exp telehealth/ or exp Internet/ or exp telecommunication/ or exp health service/

43. 10 or 42

44. 19 and 22 and 43

45. limit 44 to (english language and yr="2000 - 2011")

46. exp telemedicine/ or exp telehealth/ or Internet/ or exp telecommunication/ or health service/

47. exp telemedicine/ or exp health care policy/ or exp telehealth/ or exp Internet/ or exp telecommunication/

48. 19 and 22 and 46

49. limit 48 to (english language and yr="2000 - 2011")

50. 19 and 22 and 47

51. limit 50 to (english language and yr="2000 - 2011")

52. 10 or 46

53. 10 or 47

54. 19 and 22 and 52

55. limit 54 to (english language and yr="2000 - 2011")

56. 19 and 22 and 53

57. limit 56 to (english language and yr="2000 - 2011")

**CAB Abstracts (February 13^th^ 2014)**

1. exp stomach cancer/ or exp testicular cancer/ or exp nasal cancer/ or exp cervical cancer/ or exp pancreatic cancer/ or exp brain cancer/ or exp kidney cancer/ or exp bone cancer/ or exp laryngeal cancer/ or exp intestinal cancer/ or exp penile cancer/ or exp ovarian cancer/ or exp pharyngeal cancer/ or exp lung cancer/ or exp "head and neck cancer"/ or exp oral cancer/ or exp Deinocerites cancer/ or exp skin cancer/ or exp tongue cancer/ or exp duodenal cancer/ or exp nasopharyngeal cancer/ or exp bladder cancer/ or exp oesophageal cancer/ or exp gall bladder cancer/ or exp prostate cancer/ or exp Cancer pagurus/ or exp uterine cancer/ or exp breast cancer/ or exp colorectal cancer/ or exp endometrial cancer/ or exp International Agency for Research on Cancer/ or exp colon cancer/ or exp Cancer/ or exp thyroid cancer/ or exp liver cancer/ or exp Cancer magister/

2. cancer.tw.

3. (techniques or risk factors or surveys or breast cancer or nutritional state or human diseases or surgery).sh. or man.od. or postoperative complications.sh. or breast.sh. or men.sh. or neoplasms.sh. or postoperative care.sh. or survival.sh. or surgical operations.sh.

4. follow?up.tw.

5. (mortality or patients or breast cancer or "quality of life" or disease course or surgery or complications or morbidity or human diseases).sh. or man.od.

6. after?care.tw.

7. (diagnosis or human diseases or medicine or health care or computers or telecommunications or conferences or diagnostic techniques or information technology).sh. or man.od. or exp telemedicine/ or "cost benefit analysis".sh. or internet.sh.

8. tele?medicine.tw.

9. (health care or telemedicine or telecommunications or medical services or internet).sh. or man.od. or medical treatment.sh. or health services.sh. or information science.sh. or human diseases.sh. or health programs.sh. or biotechnology.sh. or costs.sh. or training.sh. or rural areas.sh. or health education.sh. or medicine.sh. or information technology.sh.

10. tele?health$.tw.

11. technology.tw.

12. exp traditional technology/ or exp educational technology/ or exp technology/ or exp information technology/ or exp environmental technology/ or exp construction technology/ or exp appropriate technology/ or exp technology transfer/

13. technologies.tw.

14. exp information technology/ or exp construction technology/ or exp environmental technology/ or exp traditional technology/ or exp appropriate technology/ or exp technology/ or exp technology transfer/

15. exp telecommunications/

16. exp telecommunications/

17. 1 or 2

18. 3 or 4

19. 5 or 6

20. 18 or 19

21. 7 or 8

22. 9 or 10

23. 11 or 12 or 13 or 14

24. 15 or 16

25. 17 and 20 and 21

26. limit 25 to (english language and yr="2000 - 2011")

27. 17 and 20 and 22

28. limit 27 to (english language and yr="2000 - 2011")

29. 17 and 20 and 23

30. limit 29 to (english language and yr="2000 - 2011")

31. 17 and 20 and 24

32. limit 31 to (english language and yr="2000 - 2011")

33. information technology.sh. or exp telemedicine/ or health care.sh. or computers.sh. or telecommunications.sh. or internet.sh.

34. 8 or 33

35. 17 and 20 and 34

36. limit 35 to (english language and yr="2000 - 2011")

37. (health care or telemedicine or telecommunications or medical services or internet or rural areas or medical treatment or health services or information science or medicine or information technology).sh.

38. 10 or 37

39. 17 and 20 and 38

40. limit 39 to (english language and yr="2000 - 2011")

**Medline No Revisions (10^th^ February 2014)**

1. exp Neoplasms/

2. cancer.tw.

3. exp Time Factors/ or exp Aged/ or exp Rheumatic Diseases/ or exp Adult/ or exp Arthritis, Rheumatoid/ or exp Polymers/ or exp Adolescent/ or exp Middle Aged/ or exp Follow-Up Studies/ or exp Varicocele/

4. follow?up.tw.

5. exp Aftercare/

6. after?care.tw.

7. exp Telemedicine/

8. tele?medicine.tw.

9. exp Arthroplasty, Replacement/ or exp Rural Health Services/ or exp Preoperative Care/ or exp "Diffusion of Innovation"/ or exp Evaluation Studies as Topic/ or exp Patient Education as Topic/ or exp Rural Population/ or exp Diabetes Mellitus/ or exp Telemedicine/ or exp Health Services/

10. tele?health$.tw.

11. exp Technology, Pharmaceutical/ or exp Technology Assessment, Biomedical/ or exp Fiber Optic Technology/ or exp Educational Technology/ or exp Biomedical Technology/ or exp "National Center for Health Care Technology (U.S.)"/ or exp Technology Transfer/ or exp Technology/ or exp Food Technology/ or exp Technology, High-Cost/ or exp Technology, Radiologic/ or exp Wireless Technology/ or exp "United States Office of Technology Assessment"/ or exp Technology, Dental/ or exp Green Chemistry Technology/ or exp Technology, Medical/ or exp Remote Sensing Technology/

12. exp Technology, Pharmaceutical/ or exp Technology Assessment, Biomedical/ or exp Fiber Optic Technology/ or exp Educational Technology/ or exp Biomedical Technology/ or exp "National Center for Health Care Technology (U.S.)"/ or exp Technology Transfer/ or exp Technology/ or exp Food Technology/ or exp Technology, High-Cost/ or exp Technology, Radiologic/ or exp Wireless Technology/ or exp "United States Office of Technology Assessment"/ or exp Technology, Dental/ or exp Green Chemistry Technology/ or exp Technology, Medical/ or exp Remote Sensing Technology/

13. technology.tw.

14. technologies.tw.

15. exp Telecommunications/

16. tele?communication$.tw.

17. 1 or 2

18. 3 or 4

19. 5 or 6

20. 18 or 19

21. 7 or 8

22. 9 or 10

23. 11 or 12 or 13 or 14

24. 15 or 16

25. 17 and 20 and 21

26. limit 25 to (english language and yr="2000 - 2011")

27. 17 and 20 and 22

28. limit 27 to (english language and yr="2000 - 2011")

29. 17 and 20 and 23

30. limit 29 to (english language and yr="2000 - 2011")

31. 17 and 20 and 24

32. limit 31 to (english language and yr="2000 - 2011")

33. exp Rural Population/ or exp Telemedicine/ or exp Rural Health Services/ or exp "Diffusion of Innovation"/ or exp Health Services/

34. exp Rural Population/ or exp Telemedicine/ or exp Rural Health Services/ or exp "Diffusion of Innovation"/ or exp Evaluation Studies as Topic/ or exp Health Services/

35. exp Rural Population/ or exp Telemedicine/ or exp Rural Health Services/ or exp Health Services/

36. 17 and 20 and 33

37. 17 and 20 and 34

38. 17 and 20 and 35

39. exp Rural Population/ or exp Telemedicine/ or exp Rural Health Services/

40. exp Telemedicine/ or exp Rural Health Services/

41. 10 or 40

42. 17 and 20 and 41

43. limit 42 to (english language and yr="2000 - 2011")

44. 10 or 39

45. 17 and 20 and 44

46. limit 45 to (english language and yr="2000 - 2011")

**Medline Non Indexed and In-Process Citations (1948-2011) (10^th^ February 2014)**

1. exp Neoplasms/

2. cancer.tw.

3. exp Adult/ or exp Follow-Up Studies/

4. follow?up.tw.

5. exp Aftercare/

6. after?care.tw.

7. exp Telemedicine/

8. tele?medicine.tw.

9. exp Self Care/ or exp Telecommunications/ or exp Telemedicine/ or exp Rural Population/ or exp Computer Communication Networks/ or exp Internet/

10. tele?health$.tw.

11. exp Technology Assessment, Biomedical/ or exp Technology, High-Cost/ or exp Remote Sensing Technology/ or exp Technology, Medical/ or exp Technology Transfer/ or exp Wireless Technology/ or exp Educational Technology/ or exp Biomedical Technology/ or exp Technology/

12. technology.tw.

13. exp Technology Assessment, Biomedical/ or exp Technology, High-Cost/ or exp Remote Sensing Technology/ or exp Technology, Medical/ or exp Technology Transfer/ or exp Wireless Technology/ or exp Educational Technology/ or exp Biomedical Technology/ or exp Technology/

14. technologies.tw.

15. exp Telecommunications/

16. tele?communication$.tw.

17. 1 or 2

18. 3 or 4

19. 5 or 6

20. 18 or 19

21. 7 or 8

22. 9 or 10

23. 11 or 12 or 13 or 14

24. 15 or 16

25. 17 and 20 and 21

26. limit 25 to (english language and yr="2000 - 2011")

27. 17 and 20 and 22

28. limit 27 to (english language and yr="2000 - 2011")

29. 17 and 20 and 23

30. limit 29 to (english language and yr="2000 - 2011")

31. 17 and 20 and 24

32. limit 31 to (english language and yr="2000 - 2011")

**All EBM Reviews (February 6^th^ 2014)**

1. cancer.mp. [mp=ti, ot, ab, tx, kw, ct, sh, hw]

2. neoplasm$.mp. [mp=ti, ot, ab, tx, kw, ct, sh, hw]

3. cancer.tw.

4. neoplasm$.tw.

5. follow?up.mp. [mp=ti, ot, ab, tx, kw, ct, sh, hw]

6. follow?up.tw.

7. after?care.mp. [mp=ti, ot, ab, tx, kw, ct, sh, hw]

8. after?care.tw.

9. tele?medicine.mp. [mp=ti, ot, ab, tx, kw, ct, sh, hw]

10. tele?medicine.tw.

11. tele?health$.mp. [mp=ti, ot, ab, tx, kw, ct, sh, hw]

12. tele?health$.tw.

13. technolog$.mp. [mp=ti, ot, ab, tx, kw, ct, sh, hw]

14. technolog$.tw.

15. tele?communication$.mp. [mp=ti, ot, ab, tx, kw, ct, sh, hw]

16. tele?communication.tw.

17. 1 or 2 or 3 or 4

18. 5 or 6

19. 7 or 8

20. 18 or 19

21. 9 or 10

22. 11 or 12

23. 13 or 14

24. 15 or 16

25. 17 and 20 and 21

26. 17 and 20 and 22

27. 17 and 20 and 23

28. 17 and 20 and 24
